# Integration of decoy domains derived from protein targets of pathogen effectors into plant immune receptors is widespread

**DOI:** 10.1111/nph.13869

**Published:** 2016-02-05

**Authors:** Thomas Kroj, Emilie Chanclud, Corinne Michel‐Romiti, Xavier Grand, Jean‐Benoit Morel

**Affiliations:** ^1^ INRACIRADSupAgroUMR BGPI INRA/CIRAD/SupAgroCampus International de BaillarguetTA A 54/K34398MontpellierFrance; ^2^ Université Montpellier2 Place Eugène Bataillon34095Montpellier Cedex 5France

**Keywords:** BED domain, decoy, genome, nucleotide‐binding and leucine‐rich repeat domain (NLR), plant immunity

## Abstract

- Plant immune receptors of the class of nucleotide‐binding and leucine‐rich repeat domain (NLR) proteins can contain additional domains besides canonical NB‐ARC (nucleotide‐binding adaptor shared by APAF‐1, R proteins, and CED‐4 (NB‐ARC)) and leucine‐rich repeat (LRR) domains. Recent research suggests that these additional domains act as integrated decoys recognizing effectors from pathogens. Proteins homologous to integrated decoys are suspected to be effector targets and involved in disease or resistance.
- Here, we scrutinized 31 entire plant genomes to identify putative integrated decoy domains in NLR proteins using the Interpro search. The involvement of the Zinc Finger–BED type (ZBED) protein containing a putative decoy domain, called BED, in rice (*Oryza sativa*) resistance was investigated by evaluating susceptibility to the blast fungus *Magnaporthe oryzae* in rice over‐expression and knock‐out mutants.
- This analysis showed that all plants tested had integrated various atypical protein domains into their NLR proteins (on average 3.5% of all NLR proteins). We also demonstrated that modifying the expression of the *ZBED* gene modified disease susceptibility.
- This study suggests that integration of decoy domains in NLR immune receptors is widespread and frequent in plants. The integrated decoy model is therefore a powerful concept to identify new proteins involved in disease resistance. Further in‐depth examination of additional domains in NLR proteins promises to unravel many new proteins of the plant immune system.

## Abstract 2

See also the Commentary on this article by Malik & Van der Hoorn, **210**: 371–373.

## Introduction

In plants, disease resistance is frequently conferred by nucleotide‐binding and leucine‐rich repeat domain (NLR) proteins. Indeed, the majority of cloned dominant resistance (*R*) genes broadly used by breeders code for NLR proteins. NLRs constitute huge and highly diverse gene families in plant genomes that can be further subdivided into coil‐coil NBS‐LRRs (CNLs), which contain an N‐terminal coil‐coil (CC) domain, and TIR‐NBS‐LRRs (TNLs), which have a Toll/interleukin‐1 (TIR) domain and are absent from monocot genomes (Takken & Goverse, 2012). NLR proteins act as immune receptors that recognize pathogen effectors in the cytosol. Recognition can occur directly via physical binding of the effector or indirectly via the detection of modifications that effectors cause on plant target proteins or mimics of these targets named decoys. The vast majority of the effector targets identified to date act in plant immunity, and all levels of the immune response such as signal perception, signal transduction, regulation of gene expression and defense execution are targeted by effectors. Accordingly, effector targets are involved in a broad range of cellular pathways and have many different molecular functions, such as protein kinases, transcription factors and proteases (Martin & Kamoun, 2011; Deslandes & Rivas, 2012; Presti *et al*., 2015). A third intermediate mode of effector recognition relies on the integration of decoy domains mimicking effector target proteins into NLRs (Césari *et al*., 2014; Nishimura *et al*., 2015; Wu *et al*., 2015b). Integration of decoy domains was first suspected in poplar *(Populus trichocarpa)*, where 32 NLR proteins were found to carry the same additional domain (Germain & Séguin, 2011). This additional domain, called BED (for BEAF and DREF Drosophila proteins containing this domain; Aravind, 2000), was also found in nine rice (*Oryza sativa*) NLRs, including one that was found to code for the Xa1 functional resistance protein (Das *et al*., 2014). The authors of that study speculated about why both rice and poplar have independently acquired this gene architecture and raised the hypothesis that the integrated domain may act as a sensor for pathogen effectors.

Experimental support for the integrated decoy model has been provided in the rice blast model system. The RATX1/HMA domain in the rice R proteins RGA5 and Pik‐1, which resembles the product of the blast susceptibility gene *Pi21*, interacts physically with and confers specificity to the *Magnaporthe oryzae* effectors AVR‐Pia and AVR‐Pik (Kanzaki *et al*., 2012; Cesari *et al*., 2013; Césari *et al*., 2014). Recent studies on the *Arabidopsis thaliana* NLR RRS1 also came to the conclusion that the WRKY domain integrated into RRS1 acts as an integrated decoy that recognizes the effectors AvrRps4 and PopP2 by monitoring the perturbation they induce in WRKY transcription factors that are major regulators of plant defense (Le Roux *et al*., 2015; Sarris *et al*., 2015).

By specifically analyzing the C‐terminal region of homologs of RGA5 and RRS1 from different plant species, a huge number of NLRs was identified that carry noncanonical domains frequently related to signal transduction, regulation of transcription and defense responses (Césari *et al*., 2014). This observation suggested that integration of additional domains could be a common feature of plant NLRs and be of great value in the identification of novel actors in plant immunity that may represent targets for pathogen effectors. In this study, we addressed more systematically the questions of whether the integration of additional domains in plant NLRs is a general feature and occurs in all plant lineages, which fraction of plant NLRs carry integrated domains that may act as effector sensors, and which type of domains are integrated and at what frequencies.

We show, by exploring large sets of plant NLRs from 31 genomes, that unusual domains are integrated with a mean frequency of 3.5% into NLRs. NLRs carrying unusual domains are present in all analyzed plant lineages, including mosses, and correspond to all major groups of NLRs. A huge number of functionally diverse domains were found in NLRs and they are integrated at different positions, indicating that the integration of unusual domains has occurred frequently and repeatedly during plant evolution. All these features support the model that these unusual domains in NLRs represent integrated decoys that allow plants to detect pathogen effectors that target other proteins carrying such domains. By analyzing the rice ZBED protein containing the BED zinc finger domain frequently integrated into NLRs, we confirmed that integrated decoys can be used to identify of novel players in plant immunity and confirmed a role of ZBED in rice blast resistance.

## Materials and Methods

### *In silico* analysis of NLR protein domains

To analyze a representative sample of NLR proteins from all available plant genomes, we used the NLR repertories defined by the Greenphyl database (http://www.greenphyl.org/; Conte *et al*., 2008; Rouard *et al*., 2011). Using the tribemcl software, Greenphyl has initially produced gene family clusters for rice and Arabidopsis that have been manually curated (Conte *et al*., 2008). The protein sequences of other plant genomes were allocated to these protein clusters using BlastP (Rouard *et al*., 2011) and Interpro annotation was finally included for each protein. Thus, Greenphyl has the advantage of providing gene cluster families that were defined in a unified manner across the plant kingdom. We retrieved the Greenphyl protein clusters and the corresponding Interpro domains corresponding to *c*. 155 000 NLR proteins (GP000012, GP015056, GP015065 and GP015132) as well as > 2500 receptor‐like kinases (RLKs) (GP039790). It is noteworthy that the Interpro database contains several, different, accessions for a given molecular function (see, for instance, kinase and leucine‐rich repeat (LRR)).

The criterion used to select canonical NLRs (Supporting Information Table S1) was the presence of both the NB‐ARC domain IPR002182 and an LRR domain (any of IPR001611, IPR011713, IPR003591, IPR025875, IPR006553, IPR026906, IPR008615, IPR021929, IPR013210 and IPR013101). According to the same logic, a set of 1393 canonical RLKs (Table S2) was defined by the presence of an LRR domain (same Interpro domains as above) and the protein kinase domain IPR000719. Other domains were frequently found in the NB‐ARC parts of the NLR proteins (AAA+ ATPase domain IPR003593) or the kinase domain of LRR‐RLK proteins (IPR000719, IPR008271, IPR002290, IPR017441, IPR001245 and IPR020635); these frequently found domains were therefore considered as usual components of these proteins and not as putative decoys (see Tables S2, S3).

For re‐annotation of cloned R proteins, we used the Interpro database (http://www.ebi.ac.uk/interpro/search/sequence-search) or the NCBI domain (http://www.ncbi.nlm.nih.gov/Structure/cdd/wrpsb.cgi) search tools. The gene ontology (GO) terms (http://www.ebi.ac.uk/interpro/download.html) were assigned (or manually when no GO term was available for some Interpro domains) to each Interpro domain to establish the global molecular function of decoy domains (Table S3).

### Over‐expressor and knock‐out mutant production

The ZBED cDNA from *Oryza sativa* cv Nipponbare (L.) was PCR amplified (Fig. S1b) and cloned into the pBIOS2300OX transformation vector (provided by J. C. Breitler, CIRAD) under the control of the constitutive maize (*Zea mays*) ubiquitin promoter (Fig. S1d). Nipponbare and Kitaake cultivars were transformed as described previously (Grand *et al*., 2012). For Nipponbare, the number of T‐DNA insertions was estimated in the T1 and T2 families using PCR for the geneticyn/kanamycin selection marker (Fig. S1d) as a diagnostic for the presence of T‐DNA. Only lines carrying single copy insertions (showing 3 : 1 positive PCR; *n *>* *20 plants analyzed) were conserved for further analysis. Siblings that were negative by PCR (thus not containing the transgene) were used as controls (termed null‐segregant (NS) controls). Individual T2 plants (homozygous over‐expressor and NS) from three independent monolocus lines were selfed and *ZBED* over‐expression was confirmed in the T3 progeny. In the case of transgenic plants in the Kitaake background (Fig. S2), transformation was also carried out using the pBIOS2300OX empty vector in parallel with the *ZBED* over‐expression vector. The expression of the *ZBED* gene was measured in the first generation (T0: Fig. S2a) and T1 seeds were amplified. For both vectors, T1 plants were selected on genetycin and then inoculated (Fig. S2b,c).

For the knock‐out (KO) mutant, we retrieved the ASFH06 insertion line (Nipponbare background) from the OryzaTagline collection (Larmande *et al*., 2008) (Fig. S1a). Primers flanking the insertion site were used to identify homozygous plants (*ko‐zbed*) as well as siblings showing no insertion (wt‐zbed).

### Gene expression analysis and disease resistance assays

Rice plant growth and inoculation were performed as described previously (Vergne *et al*., 2010). Three‐week‐old plants were inoculated with the moderately virulent isolate GUY11 of *M. oryzae*. Five to 7 d after inoculation, susceptible lesions (spots characterized by a grayish center) were counted as a measure of susceptibility. Differences in total lesion numbers between Fig. 3(b) and 3(e) only reflect differences in inoculum quality.

RNA and gene expression was determined using qRT‐PCR (expression data were normalized with the *Actin* gene) were done as described in Grand *et al*. (2012). The qRT‐PCR primers used for ZBED (accession LOC_Os01g36670) were CCGTTGATTCAGAGGTGCTGAC and TCGTAACAACATGGTAGCCTTGG.

## Results

### Hypothetical integrated decoy domains are found in 15% of the cloned NLR R proteins

**Figure 1:**
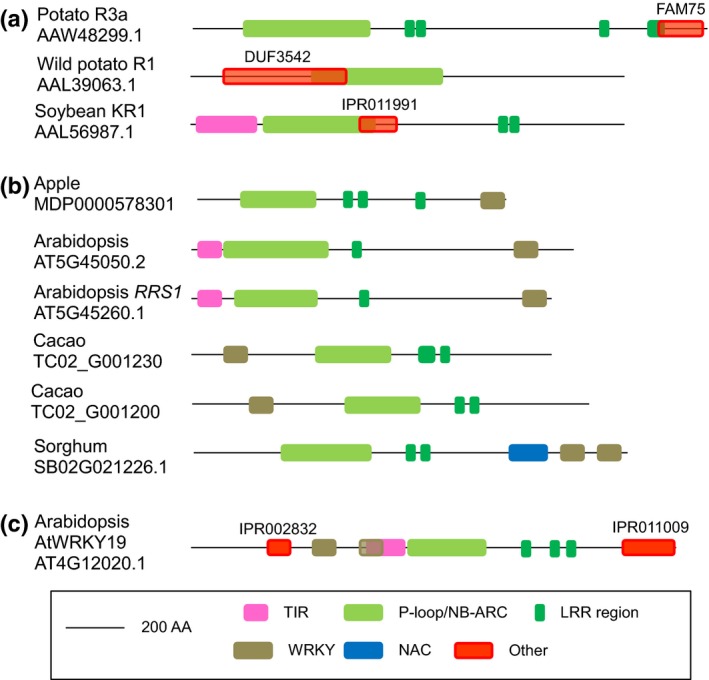
Examples of integration of unusual domains in nucleotide‐binding and leucine‐rich repeat (NLR) proteins in plants. Protein domains of the different NLRs were established by an InterPro search (http://www.ebi.ac.uk/interpro/) or NCBI domain search (http://www.ncbi.nlm.nih.gov/Structure/cdd/wrpsb.cgi) along the full‐length protein (black line). The Interpro search does not detect coil–coil domains often found in NLRs, thus explaining their absence here. Canonical NLR domains (Tol Interleukin Receptor (TIR), P‐loop/NB‐ARC and LRR) are indicated in pink and green. Although these domains may be larger, the detection by the Interpro search only indicates the borders defined by the Interpro domains. (a) The R3a, R1 and KR1 cloned resistance proteins contain unusual protein domains besides the canonical NLR structure. In KR1, IPR011991 represents a winged helix‐turn‐helix DNA‐binding domain, whereas FAM75 and DUF3542 are domains of unknown function. (b) The WRKY domain (brown) was integrated several times in plant NLR genes from different species and some examples are shown here. An additional NAC domain is also found in the sorghum protein. (c) The *Arabidopsis thaliana* NLR At4g12020 is highly modular and contains three types of additional domains besides the canonical TIR‐NBS‐LRR domains: a WRKY domain, a PAH domain of unknown function (IPR002832) and a kinase‐like domain (IPR011009).

First, we retrieved the sequences of cloned R proteins and expert‐annotated R protein analogs of the NLR class from the Plant Resistance Gene Data Base (PRGdb) (Sanseverino *et al*., 2013; Table S4) and searched this data set of 98 NLRs for additional Interpro domains not present in canonical NLRs (see the Materials and Methods section). In addition to the nine previously reported R proteins with putative decoy domains (Césari *et al*., 2014) (Table S1), there are three known R proteins with putative decoy domains: the auto‐immune NLR CHS3, which has a LIM‐type zinc‐finger domain (Bi *et al*., 2011), the major splice variant of the rice blast R protein Pi‐ta containing a thioredoxin domain (Costanzo & Jia, 2009), and the rice bacterial blight resistance protein Xa1 which contains a BED‐type zinc‐finger domain that was overlooked when Xa1 was cloned (Yoshimura *et al*., 1998) and was also described in several recent studies (Germain & Séguin, 2011; Das *et al*., 2014). Moreover, we identified three R proteins with additional domains that had not yet been detected (Fig. 1a): the potato (*Solanum tuberosum*) late blight resistance protein R3a carries a FAM75 domain of unknown function in its C terminus; the R1 protein from a wild potato (*Solanum demissum*) species contains a domain of unknown function; and the putative soybean (*Glycine max*) R protein KR1 (He *et al*., 2003) contains a DNA‐binding domain. Thus, in total, 15 of the 98 functional R proteins of the NLR class contain a putative decoy domain, indicating that up to 15% of the NLRs acting as R proteins contain integrated decoy domains.

### Integrated domains are present in NLR proteins from 31 plant genomes

The analysis of cloned R proteins and previous reports suggest that NLRs with integrated decoy domains may be common in many plant genomes (Césari *et al*., 2014). To test this hypothesis in a global manner, we analyzed the NLR repertoires in 30 complete genomes from higher plants and one moss species provided by the Greenphyl database which generated protein family clusters from plant whole‐genome sequences by using Tribe MCL and Blast similarity searches (for details see the Materials and Methods section; Rouard *et al*., 2011). The Greenphyl database records slightly more than 15 500 NLRs in these 31 plant genomes belonging to either the TNL orthology group GP000012 or one of the three major CNL orthology groups GP015065, GP015132 or GP015056. This set of NLRs has the advantage that the same criteria (see the Materials and Methods section) were used to retrieve proteins from whole‐genome data for large and complex gene families. Comparison of the Greenphyl NLR sets provided to the manually curated NLR repertoires from rice (Luo *et al*., 2012) and *A. thaliana* (Tan *et al*., 2007) showed that the Greenphyl repertoire contained 236 of the 276 published and manually curated rice NLRs (85%) and 177 of the 201 manually curated *A. thaliana* NLRs (88%). From this, we concluded that the NLR repertoires from Greenphyl provide extensive coverage of the NLR diversity in plant genomes.

Conversely, we observed that the Greenphyl NLR data set contained rice and *A. thaliana* proteins not present in the previously published curated NLR lists (Tan *et al*., 2007; Luo *et al*., 2012). This is partially explained by the fact that the Greenphyl NLR data set contains proteins carrying either the LRR or the NB‐ARC domain but not both of them. Therefore, we also selected among the more than 15 500 NLRs recorded by Greenphyl a subset of 2699 canonical NLRs based on the *combined* presence of the NB‐ARC and the LRR Interpro domains (Table S1).

**Figure 2:**
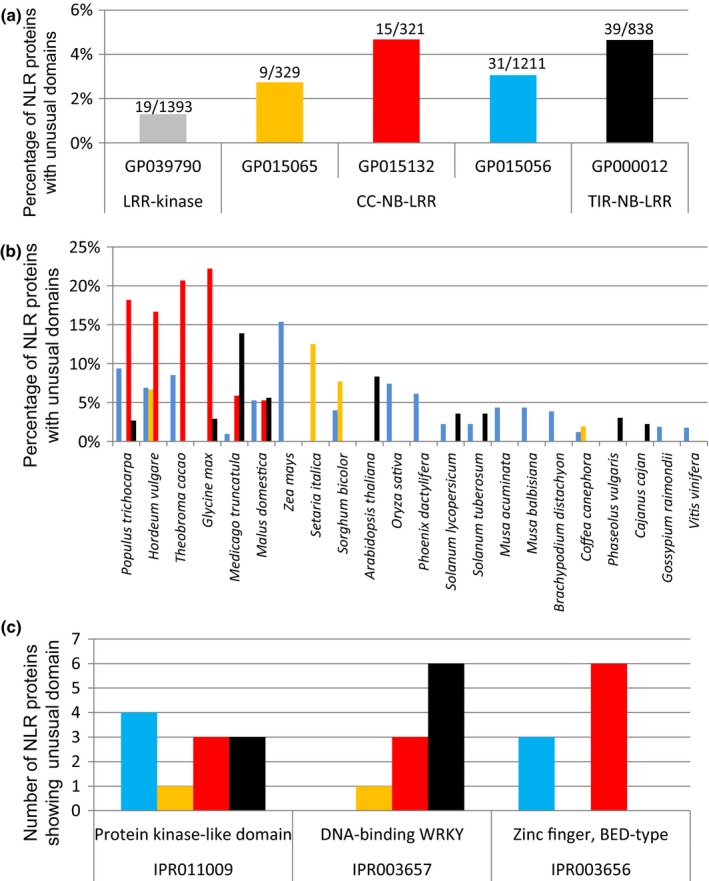
Most plant species show unusual domains in their nucleotide‐binding and leucine‐rich repeat (NLR) proteins. Additional domains, according to Interpro annotations, were searched in 2699 plant NLR proteins from different families of canonical NLRs. The NLR subgroups defined by Greenphyl are shown with different colors: orange (GP015065), red (GP015132), blue (GP015056) and black (TIR‐NB‐LRR: GP000012). (a) The number of unusual domains and the number of NLRs analyzed is indicated above each bar (see Supporting Information Table S1 for more detail). The 1393 canonical receptor‐like kinases (RLKs) from Greenphyl family GP039790 (gray bars) were used as a control to evaluate, in this different set of multi‐domain immune receptor proteins, the frequency of such unusual domains (see Table S2 for more detail). (b) The 22 plant genomes showing the highest frequencies and (c) the number of NLR proteins carrying the most frequent domains (IPR011009, IPR003657 and IPR003656) are shown.

We retrieved the Interpro annotations for the *c*. 15 500 NLR proteins and searched for domains that did not correspond to canonical CNL or TNL domains. This identified 456 NLR proteins (3.6%) harboring at least one unusual domain (Fig. S3). The same analysis was performed with the more stringently defined data set of 2699 canonical NLRs. This identified 94 NLRs (3.5%) with unusual domains (Fig. 2a), a value very similar to that obtained with the full Greenphyl set of more than 15 500 NLRs.

The two major classes of NLRs showed slight differences, with CNLs displaying fewer unusual domains (2.5%) than TNLs (4.7%). In addition, the frequency of NLRs with unusual domains varied between species (Fig. 2a), with a maximum of 6% in poplar. Among the 10 NLRs of the moss *Phycomistrella patens*, two displayed an integrated domain, indicating that this phenomenon extends to lower plants.

As many genomes are machine predictions, erroneous annotations leading to gene fusions could produce artefactual integrated domains. We thus tested whether another class of plant receptor, the RLKs, displayed a similar occurrence of unusual domains. Out of *c*. 1400 RLK proteins tested, only 19 proteins (1.4%) displayed additional domains that did not correspond to canonical domains for RLK proteins (Fig. 2a; see the Materials and Methods section and Fig. S3). Although the frequency of additional domains in NLRs was two‐fold higher than in RLK proteins, some of the integrated domains in NLRs could still have resulted from wrong genome annotations, such as gene fusion. Taken together, the genomes of all 31 analyzed higher plants contained significant but varying numbers of NLR proteins with additional unusual domains.

### Kinase, WRKY and BED domains are most frequent among the myriad molecular functions hidden in NLR proteins

Automatic gene ontology assignment and manual annotation of the 94 Interpro domains identified in the 2699 canonical NLRs identified some molecular functions that were highly represented; that is, nucleic‐acid binding activity (including transcription), signaling and oxidative metabolism (Table S3). However, most of the domains belonged to other very diverse classes that could not be regrouped, indicating that integrated domains encompass very diverse molecular functions.

Most domains occurred at low frequencies and were often detected only once. Only three domains were found in many NLRs and in different species (Fig. 2c): the protein kinase domain IPR011009 involved in protein phosphorylation, the WRKY domain IPR003657 involved in DNA‐binding and transcription, and the BED domain IPR003656 involved in DNA‐binding. These domains were found 11, 10 and nine times and in six, four and four different species, respectively. A particular feature of the BED domain is that it is particularly frequent in the N‐terminus part of NLRs, as described previously in poplar (Germain & Séguin, 2011).

### Uncommon domains have been integrated frequently and independently into NLRs during plant evolution

Acquisition of domains by NLRs seems to have occurred several times and independently during evolution, as the same domain is present in NLRs from different phylogenetic clades. For instance, WRKY (IPR003657) domains were found in CNLs of the groups GP015132 and GP015056 and in TNLs of group GP000012 (Fig. 2b; Table S1). Further support for repeated and independent acquisition of integrated domains comes from the observation that the same domain can be integrated at different locations. WRKY domains are, for instance, detected in the N‐ or the C‐terminus of CNLs and the C‐terminus of TNLs (Fig. 1b). This apparent flexibility in the integration of additional domains in NLRs had already been reported for the integration of the RATX1/HMA domain in the rice blast resistance NLRs RGA5 and Pik‐1 where it is located, respectively, at the C‐terminus or between the CC and the NB domains (Kanzaki *et al*., 2012; Cesari *et al*., 2013).

In rare cases, several domains were integrated in one single NLR. For example, one sorghum (*Sorghum bicolor*) CNL contains a NAC and two WRKY domains (Fig. 1b) and an *A. thaliana* TNL (AT4G12020, also known as AtWRKY19; Fig. 1c) carries two WRKY domains, one kinase domain (IPR001109) and one PAH domain (IPR002832) involved in protein–protein interactions. Taken together, these examples suggest a high modularity of the NLR proteins.

### A protein containing three BED domains affects disease resistance

**Figure 3:**
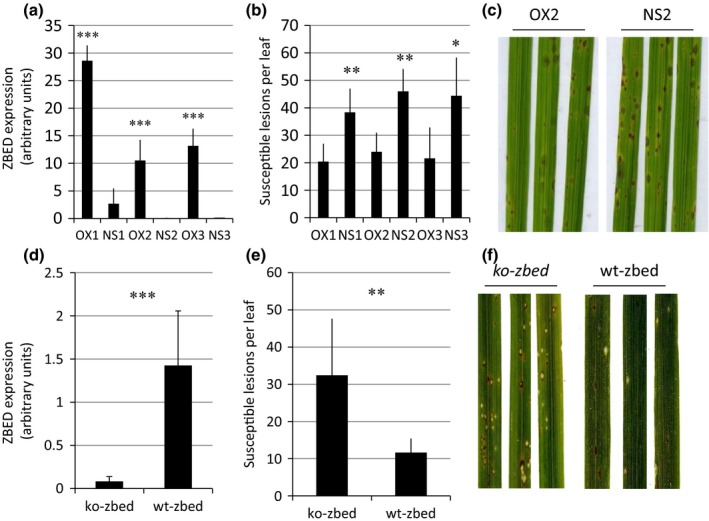
The ZBED protein is involved in blast resistance. The role of the rice ZBED protein containing three BED domains in disease resistance against the blast fungus *Magnaporthe oryzae* was evaluated by over‐expressing (Supporting Information Fig. S1b–d) and knocking out (Fig. S1a) the *ZBED* gene. The three homozygous, monolocus T3 transgenic rice lines over‐expressing the *ZBED* gene (a) showed fewer disease lesions (b, c). By contrast, the insertion mutant for the *ZBED* gene (d) showed more disease symptoms (e, f). *ZBED* gene expression was measured by qRT‐PCR (normalized by *Actin*). Disease lesions, characterized by sporulation and a grayish center, were quantified 7 d after inoculation with the *M. oryzae* strain GUY11 and representative symptoms are shown (c, f). Such differences in symptom strength between (c) and (f) are frequent in this type of plant–pathogen interaction study and are probably attributable to variability in plant growth conditions. A Student *t*‐test was used to compare the over‐expresser lines (OX) and the knock‐out (ko) mutant line with their respective null‐segregant controls (NS; see the Materials and Methods section): *, *P *<* *0.05, **, *P *<* *0.01; ***, *P *<* *0.001. The gene expression and disease values are the mean + SD from three independent experiments.

The integrated decoy hypothesis predicts that some plant proteins similar to decoy domains are targeted by effector proteins because they act in disease resistance or susceptibility. The decoy domains in NLRs could therefore guide the identification of novel effector targets and new proteins involved in plant immunity. We challenged this hypothesis for the BED domain, which is frequently integrated in NLRs but had, thus far, not been demonstrated to play a role *per se* in disease resistance. For this purpose, we investigated the ZBED protein from rice, which contains three BED domains (Fig. S4a) that show 44% to 58% identity with the BED domains of Xa1 (Fig. S4b). *ZBED* had attracted our attention because its expression is negatively correlated to partial resistance to the blast fungus *M. oryzae* across a panel of rice varieties (Fig. S2c). This type of expression pattern has been reported to be a hallmark of regulators of disease resistance (Grand *et al*., 2012). The *ZBED* gene is not strongly regulated during infection by the rice blast fungus *M. oryzae* (Fig. S4d). To investigate whether ZBED has a role in immunity, rice plants over‐expressing the *ZBED* gene were produced. These plants did not show any obvious visible growth phenotype (data not shown); however, rice plants of the variety Nipponbare over‐expressing *ZBED* (Fig. 3a) were less susceptible to *M. oryzae* and developed fewer disease lesions than control plants (Fig. 3b,c). This observation could be confirmed in a separate genetic background, Kitaake (Fig. S2), further supporting a role of ZBED in disease resistance. There was a weak correlation between transgene expression levels and disease symptoms (compare Fig. 3a with 3b, and Fig. S2a with S2c). As over‐expression of many genes may artefactually lead to reduced susceptibility, we also analyzed *ZBED* T‐DNA insertion mutants. We isolated a homozygous mutant for the *ZBED* gene (*ko‐zbed*) as well as homozygous wild‐type siblings (*wt‐bzed*) (Fig. S1a) and inoculated them with *M. oryzae*. Loss of ZBED expression led to a significant increase in susceptibility (Fig. 3e,f), indicating that ZBED contributes to resistance to the rice blast fungus *M. oryzae* either by directly affecting fungal growth or by contributing to the induction of defense responses. These results for ZBED demonstrate that the identification of integrated decoy domains can serve as an extremely powerful way for the identification of new actors in plant immunity.

## Discussion

The discovery that unusual domains integrated into NLR immune receptors act as decoy domains is very recent. The first experimental evidence was provided by the investigation of the rice blast resistance NLR pairs RGA4/RGA5 and Pik‐1/Pik‐2 and important validation was supplied by detailed analysis of the *A. thaliana* NLR pair RPS4/RRS1 (Kanzaki *et al*., 2012; Cesari *et al*., 2013; Le Roux *et al*., 2015; Sarris *et al*., 2015). The occurrence of NLRs with decoy domains in phylogenetically unrelated immune receptors from monocot and dicot plants suggested that it is a widespread phenomenon and the first large‐scale analysis showed that the integration of unusual domains is frequent among cloned R proteins of the NLR class and RGA homologs similar to RGA5 and RRS1 (Césari *et al*., 2014). In the present study, we have analyzed more systematically the occurrence of integrated decoy domains in NLRs from 31 plant species: 30 higher plants and one moss species. For our systemic analysis, we used the publicly available data of the GreenPhyl database where a huge number of sequenced genomes have been analyzed with a common set of criteria for establishing protein family clusters (Rouard *et al*., 2011). Comparison of this NLR data set with the published, expert‐curated NLR repertoires of *A. thaliana* and rice showed for both species a good representativeness of the GreenPhyl database and suggested good coverage for the other species. This data set was also used to define a more stringent data set of NLRs. Both NLR data sets were filtered according to the occurrence of Interpro domains, leading to the definition of NLRs with additional, noncanonical domains. It is noteworthy that our approach using Interpro annotation has some limitations. For instance, the CNL domain found in almost half of the TIR‐LRR proteins (Dodds *et al*., 2001) was not detected in our study because this domain is not defined in the Interpro database. Similarly, the CC domain found in canonical NLRs was not detected. Despite these limitations, our analysis clearly demonstrated that decoy integration is frequent and occurs in all analyzed plant lineages. Indeed, NLRs from many different lineages of annual and perennial dicotyledonous plants, several different lineages of monocotyledonous plants and one moss possess decoy domains at varying frequencies. Decoy domains are found in all classes of NLRs, that is, in the different classes of CNLs and in TNLs, and these NLRs seem tremendously flexible in terms of the integration of decoys, as integration can occur at the boundaries of all the different canonical NLR domains, and multiple integrations at different positions in a single NLR are even observed. The *A. thaliana* TNL At4g12020 is, for instance, highly modular and contains three types of additional domains, integrated C‐ or N‐terminal to the canonical TIR, NBS and LRR domains (Fig. 1).

Our analysis also revealed that integrated decoy domains are extremely diverse and cover many different molecular activities. Out of the 90 putative decoy domains identified here, 64 are novel (Table S3) compared with the initial study by Césari *et al*. (2014). Conversely, 34 of the 52 Interpro domains found in RGA5 and RRS1 homologs were not detected in this analysis. Thus, our analysis is probably not exhaustive, and future analysis employing additional and in future more sensitive approaches for domain detection or more complete data sets, including for example additional NLRs from re‐annotated and expert‐curated genomes, targeted NLR sequencing by RenSeq (Jupe *et al*., 2013) or inclusion of additional species or more than one accession per species will further clarify the picture.

For both analyzed data sets, the more loosely defined *c*. 15 500 and the more stringently defined 2699 NLRs, our analysis revealed a mean frequency of 3.5% for unusual domain integration (Figs 1, S3). This is much lower than the frequency of decoy domain integration in cloned R proteins of the NLR class, which is 15% (Table S4). It could be that NLRs that are functional as immune receptors and confer resistance to pathogens contain more frequently integrated domains than NLRs that have no defined specificity and rather serve as a reservoir for the diversification of NLR receptors or act in processes other than pathogen recognition and disease resistance. The ongoing extensive cloning of *R* genes and the increased reliability of annotation of whole‐genome NLR complements through technical advances, such as the extremely powerful RenSeq approach, will allow this issue to be addressed more thoroughly in the near future.

Integrated decoy domains were first studied in paired NLRs and in the homologs of the paired NLRs RGA5 and RRS1 (Césari *et al*., 2014). These observations raised the question of whether decoy domains are restricted to paired NLRs. Our study has identified decoy domains in NLR type R proteins such as R3a and Xa1 that, according to current knowledge, are not paired and do not show the typical, tightly linked head‐to head tandem arrangement with a second NLR in the genome (Yoshimura *et al*., 1998; Huang *et al*., 2004). In addition, our inspection of the genes encoding NLRs with integrated decoys in rice revealed that many of them do not show the typical genomic head‐to‐head tandem organization and often even do not possess an adjacent neighboring NLR (Table S5). Therefore, integrated decoy domains seem not to be restricted to paired, matching NLRs. Whether the nonpaired NLRs that contain integrated decoy domains act in concert with downstream helper NLRs (Bonardi *et al*., 2011; Wu *et al*., 2015a) will have to be elucidated in the future.

Three domains have been identified particularly frequently in NLRs, the WRKY and BED zinc‐finger domains and the protein kinase domain. For all these three domains, cloned R proteins are known, demonstrating that the corresponding chimerical NLR structures are functional as immune receptors in effector‐mediated pathogen recognition. The WRKY domain is present in the RRS1 protein from *A. thaliana* (Deslandes *et al*., 2002), where it has been convincingly shown to act as a decoy in the recognition of the effectors PopP2 and AvrRPS4 from the bacterial plant pathogens *Ralstonia solanacearum* and *Pseudomonas syringae* pv *tomato* (Le Roux *et al*., 2015; Sarris *et al*., 2015). A protein kinase domain is present in the rust resistance protein RPG5 from *Hordeum vulgare* and in the susceptibility factor Tsn1 from wheat which confers, in an inverse gene‐for‐gene manner, susceptibility to isolates of the necrotrophic fungi *Pyrenophora tritici repentis* and *Stagonospora nodorum* carrying the ToxA effector protein (Faris *et al*., 2010; Wang *et al*., 2013b). The BED domain is present in the Xa1 resistance protein from *O. sativa* (Yoshimura *et al*., 1998) which recognizes an unknown factor from the bacterial blight pathogen *Xanthomonas oryzae* pv *oryzae*.

In addition to the three most frequently found unusual domains, domains corresponding to a wide range of molecular functions have been observed to be integrated. This is in accordance with the hypothesis that these integrated additional domains serve to monitor modifications of the complex plant immune system. Integrated domains are unlikely to act in downstream signaling triggered by NLR‐mediated effector recognition, which is thought to be rather conserved between NLRs and between species. An activity of the integrated domains in other pathways and in the absence of the Avr effector cannot be completely excluded until it is experimentally demonstrated not to occur, but seems, in particular in the cases where partial or nonfunctional domains are integrated, rather improbable (Wu *et al*., 2015b).

Some integrated domains are characteristic of well‐known key players in plant immunity such as protein kinases and WRKY transcription factors. Others have been demonstrated more recently to be involved in resistance, targeted by effectors or guarded by NLRs. NAC transcription factors have, for instance, only very recently been shown to be targeted by an effector of the potato light bight pathogen *Pythophthora infestans* and to play an important role in resistance to this pathogen (McLellan *et al*., 2013). Corresponding NAC domains are integrated in different classes of NLRs from mono‐ and dicotyledonous plants (Table S1). Many other domains, not yet implicated in immunity and disease development, are integrated in NLRs. This allows us now to take a completely new and extremely exciting look at plant immunity and disease susceptibility by picking proteins containing these decoy domains and testing whether they are involved in resistance or disease‐related processes.

The integrated decoy concept predicts that some proteins containing BED domains, the third most frequently integrated decoy domain, may be involved in disease resistance. Our analysis of the rice *ZBED* gene supports this hypothesis, as a rice *ZBED* mutant is more susceptible to rice blast while ZBED over‐expresser lines show increased resistance. This suggests that BED domains in NLRs may indeed represent decoys mimicking BED proteins involved in some as yet unknown immunity‐related processes. However, the putative effector targeting the BED domains remains to be identified. In animals, proteins with BED domains were initially described in Drosophila, where they have DNA‐binding activities (Aravind, 2000). BED proteins in animals have diverse molecular functions; for instance, the Zbed3 protein is a transcriptional regulator (Wang *et al*., 2013b) while Zbed6 is secreted to the membrane (Jia *et al*., 2014). To understand the role of BED domains in plant immunity, thorough investigation of ZBED and related BED proteins will be required.

The integrated decoy model adds one more level of complexity to our understanding of effector recognition by plant NLR immune receptors and opens exciting new avenues in the investigation of plant immunity. Exploring the role of proteins containing the domains that correspond to the integrated decoys promises to shed new light on the biological functions that phylogenetically unrelated pathogens have chosen to target during evolution.

## Author contributions

T.K. and J‐B.M. designed the research; E.C., C.M‐R., X.G. and J‐B.M. performed the research; E.C., C.M‐R., T.K. and J‐B.M. analyzed and interpreted the data; T.K., E.C. and J‐B.M. wrote the manuscript.

## Supporting information

Please note: Wiley Blackwell are not responsible for the content or functionality of any supporting information supplied by the authors. Any queries (other than missing material) should be directed to the *New Phytologist* Central Office.

**Fig. S1** *ZBED* over‐expression and knock‐out lines in the Nipponbare background.
**Fig. S2** ZBED transgenic lines (Kitaake background) are more resistant to the rice blast fungus.
**Fig. S3** Frequency of unusual domains in all Greenphyl > 15 500 NLR proteins.
**Fig. S4** Structure and expression of the ZBED protein and gene.

**Table S1** Unusual domains in canonical plant NLR proteins

**Table S2** Unusual domains in canonical plant RLK proteins

**Table S3** The 90 putative integrated decoys identified

**Table S4** Curated cloned plant R genes

**Table S5** Rice NLRs with integrated decoys and their neighborhood
