# Supplementary material for: Integration of decoy domains derived from protein targets of pathogen effectors into plant immune receptors is widespread
Source: New Phytol. 2016 Feb 5;210(2):618–26. doi: 10.1111/nph.13869 (PMC5067614; doi:10.1111/nph.13869)
Supplement: Supplementary file 1 — Fig. S1 ZBED over‐expression and knock‐out lines in the Nipponbare background. Fig. S2 ZBED transgenic lines (Kitaake background) are more resistant to the rice blast fungus. Fig. S3 Frequency of unusual domains in all Greenphyl > 15 500 NLR proteins. Fig. S4 Structure and expression of the ZBED protein and gene. [file NPH-210-618-s001.pdf]

Integration of decoy domains derived from protein targets of pathogen effectors into plant immune receptors is widespread

Kroj Thomas, Chanclud Emilie, Michel-Romiti Corinne, Grand Xavier, and Morel Jean-Benoit

Accepted Dec 16th 2015

Suppl Figure 1 Kroj et al

a.

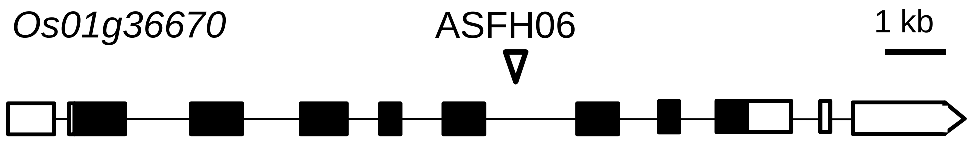

b. **ZBED protein corresponding to the cloned cDNA**

<u>MDGVDKDKGNGKVHFRDSSPQEAFRTYKRRRQPRPEPQQQPQPQPQSEPEPQQQ</u> PQ
PQPQPQPQPEAEAEAEAEAKAADVLARQVTETFWKSRDIGWKHGIMIDENRQHWKCMYCH
LTRYGGGVSRLKRHLAGDLDVKMCPKVPADVSEKIREHLRKKRERRKKRAAQNRDNCVTA
KSTSDDIKSGKDPLPVDSEVLTGVDTVLEEVTNQ TNHDNQDLTYPKATMLLRGIRDIGWE
HAVDLDGNKRRWKCKWCSLCRSGGVTTLKAHLTDSSCPNIPKEISKKVLNFIEEKRAARH
LFNSAAKSPFNVKFDEDAVNLSEIQVEGTPPLTDDRQPLGNSLHIQTSECTINEFEKVAA
GSNQQGAEHSNQLLNHGEQLMKSSDQPEEHCTLEHGRCQVLDNNKQQTMDNKTDNPEHKE
VLKHPKKTRFNIRKHIVIVDESARHWRCRYCGLDGYGKTSRLHFHLAAVFRHPKCPSVPK
EVFAKARHHIHLKRRLNVKKAGQQARSRPHILGQSSQQQQNNNPVLSNYPTRLRDNAWEH
SLIHDKEKGHWKCKWCSLEGYHGITRLKWHLVGWQNRPQCLNVPEDVAKTIRDKMISREK
QKEGRLNLDVIDSCNMPCSSESLQFDQENFAEVMQGKGSSEDFNQAERQSNTLNTVCNTT
HPPQNSNNYQGLQENGLYSSKNKSEKQTERYDCWSHWRYVLDGLMHLPGALEGPGIQSCI
RDVLLYGSAEFGTVGDKVEMDSNRKVSSDGNIAKCQSVLVDVLKSENFALLCNVLGRTVH
QDEQRTKYFDFTMIDSRMKNGDYGRAPLLFKHDLKMVTPLL*

c.

```
              1        10        20        30        40        50     57
              |--------+---------+---------+---------+---------+------|
     ZBED     MDGVDKDKGNGKVHFRDSSPQ---EAFRTYKRRRQPRPEPQQQPQPQPQSEPEPQQQ
     KOME      MVLTRIKGMGRCISGIPLPRKLSEPTRGAGSHGRSRNSNRSRSRSQSRNHSNSHSQ
Consensus     .dgldrdKGnGrchfrdplPr...EafRgagrrrrpRnenrqrprpQprnep#pqqQ
```

d.

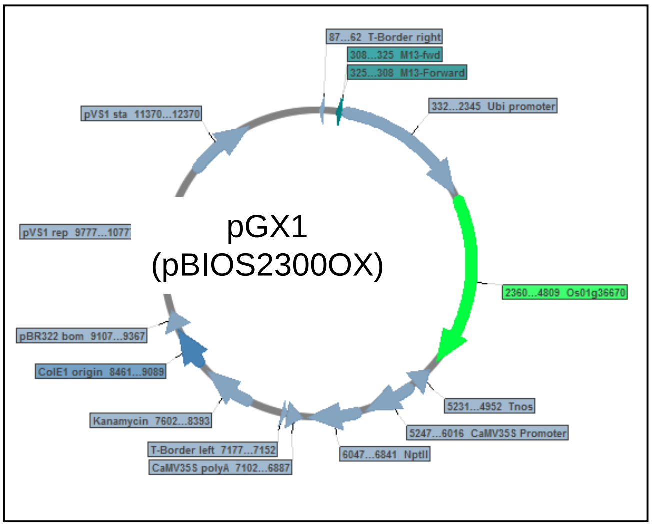

Fig. S1. *ZBED* over-expression and knock-out lines in the Nipponbare background
The genomic structure of the ZBED gene (intron/exon: white/black) is shown in (**a**). The triangle represents the position of the analyzed T-DNA insertion line (ASFH06). For over-expression, a full-length cDNA (**b**) was cloned from Nipponbare and used for transformation (see Methods). The underlined ZBED sequence cloned from rice cDNA is slightly different from the published sequence from the KOME database (**c**). The *ZBED* cDNA clone was inserted into the pBIOS2300OX vector under the control of the Ubiquitin constitutive promoter and the Kanamycin/Genetycin selection marker (**d**) as in Grand et al (2012).

Suppl Figure 2 Kroj et al

a.

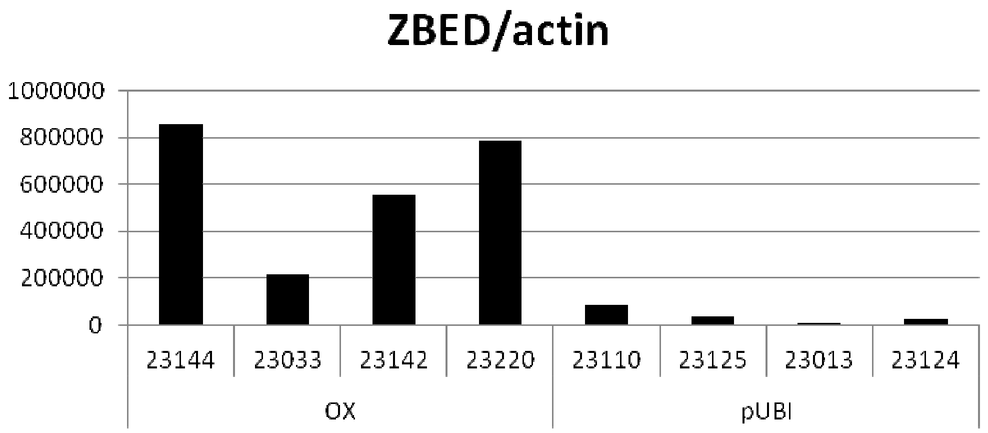

b.

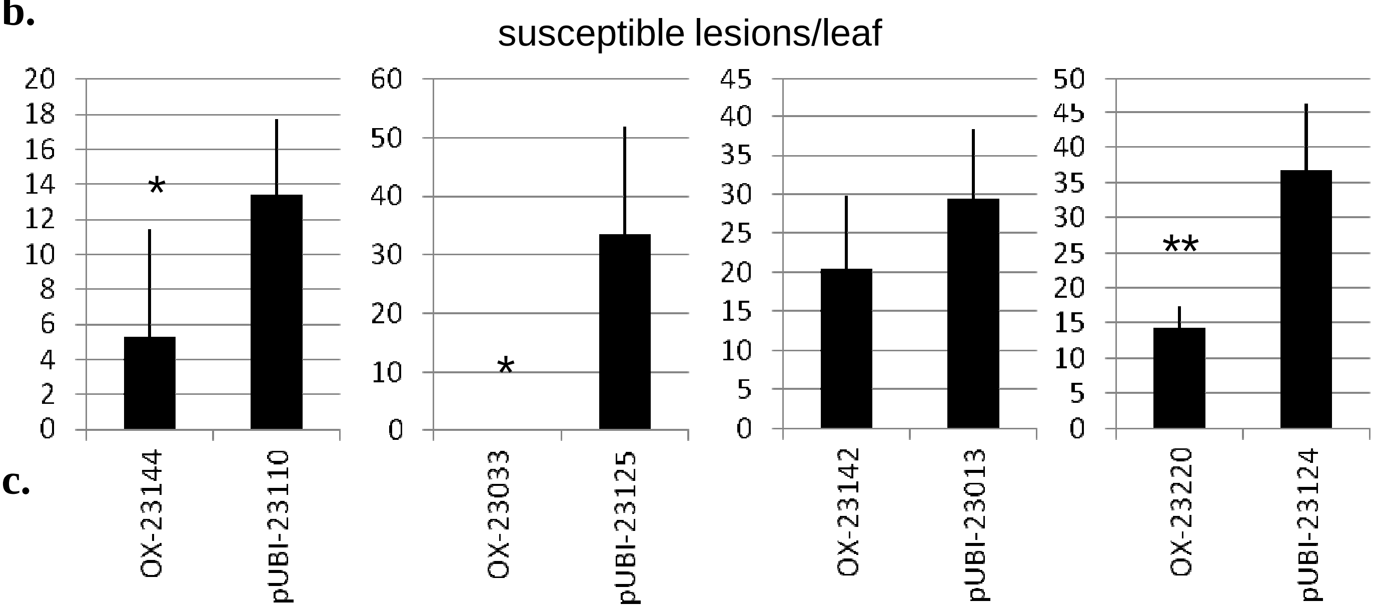

c.

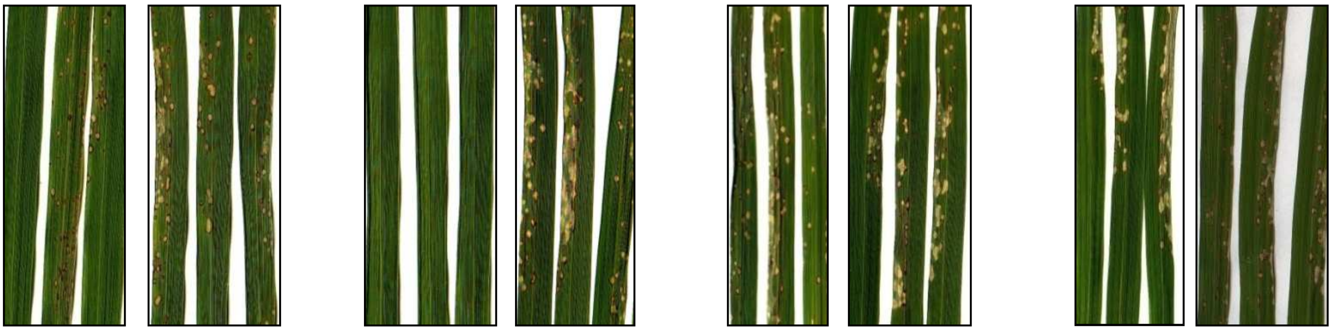

Fig. S2. ZBED-overexpressing transgenic lines (Kitaake background) are more resistant to the rice blast fungus

The vector shown Fig. S3d and the original empty vector (pUBI= pBIOS2300OX) were used to transform the rice Kitaake genotype. Individual T0 lines showing ZBED over-expression (**c**) were selected. T1 plants overexpressing *ZBED* or carrying the empty vector were sown on Kanamycin. Lines showing 3:1 Kanamycin resistance segregation (single locus insertion) were further analyzed. Pairs of Kanamycin resistant plants for *ZBED* over-expression vector and empty vector were transplanted for inoculation with the GY11 *M. oryzae* strain*.* The four pairs of *ZBED* over-expressor (OX) and empty vector (pUBI) lines were conducted independently. The ZBED gene expression (normalized with Actin) was measured on unique T0 plants (**a**). The symptoms (susceptible lesions) were counted 7 days after inoculation (**b**) and representative examples are provided (**c**). In panel (**b**), A T-test was used to compare OX and pUBI (*: P<0.05).

Suppl Figure 3 Kroj et al

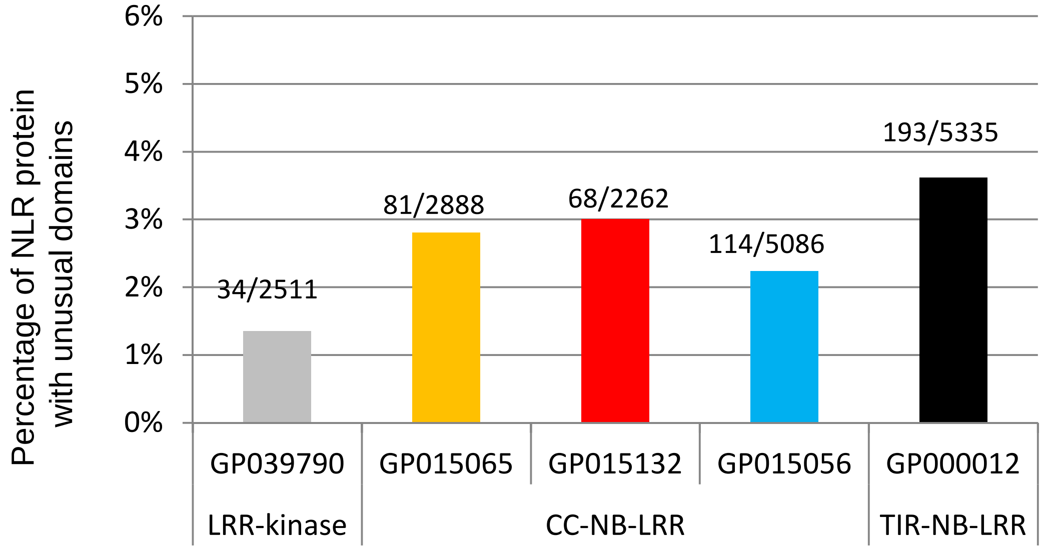

Fig. S3. Frequency of unusual domains in all Greenphyl NLR proteins

The approximately15500 NLR proteins from the Greenphyl database were analyzed (see Methods). Non-canonical, unusual domains were searched using InterPro annotation of the NLR proteins. The number of unusual domains and the number of analyzed NLRs per Greenphyl family is indicated above each bar. The 2511 RLKs from Greenphyl family GP039790 (grey bars) were used as a control and the frequency of unusual domains, other than LRR and kinase, was evaluated in this different set of multi-domain immune receptor proteins.

Suppl Figure 4 Kroj et al

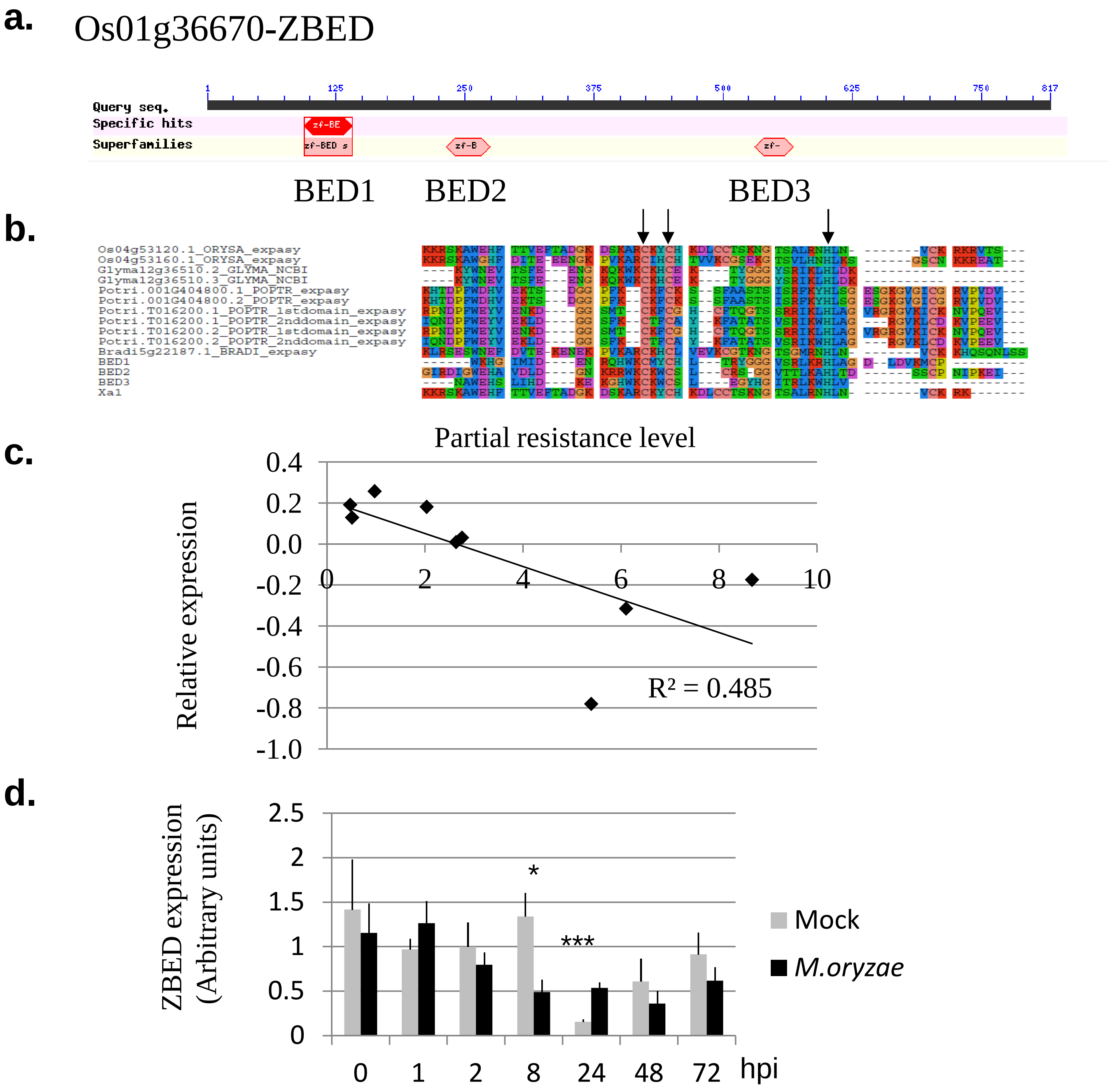

Fig. S4. Structure and expression of the ZBED protein and gene

(**a**) The ZBED protein contains three predicted BED domains (screenshot from ncbi domain search http://www.ncbi.nlm.nih.gov/Structure/cdd/wrpsb.cgi). (**b**) Alignment of the three BED domains from ZBED and the BED domain (IPR003656) identified in the Xa1 protein (Table S4 and Fig. 1a). Two Cysteine and one Histidine residues conserved across all proteins are indicated by arrows. (**c**) The constitutive expression of ZBED is negatively correlated to partial resistance level against *M. oryzae* as measured in Grand et al (2012). This measure relies on inoculations with several multivirulent isolates of M. oryzae that allow global evaluation of quantitative/partial blast resistance of a given rice variety. Each point in the graph represents this value for one rice variety for which ZBED expression was also measured. (**d**) The ZBED gene is slightly repressed by *M. oryzae* infection. The fact that the expression in mock treated plants also changes along time is classical in this type of experiments where the light regime and hygrometry are changing during the experiments (the first 8 h are in the dark for inoculation purposes). The data represent mean and SD from three independent biological experiments. A Student t-test was used to compare the infected plants with non-inoculated plants (Mock): *: P<0.05, ***: P<0.001.

References

Grand, X., Espinoza, R., Michel, C., Cros, S., Chalvon, V., Jacobs, J., and Morel, J.-B. (2012). Identification of positive and negative regulators of disease resistance to rice blast fungus using constitutive gene expression patterns. *Plant Biotechnol. J*. 10: 840–50.
